# Effect of octenidine mouthwash on plaque, gingivitis, and oral microbial growth: A systematic review

**DOI:** 10.1002/cre2.386

**Published:** 2021-07-06

**Authors:** Vishakha Grover, Jaideep Mahendra, Dharmrajan Gopalakrishnan, Ashish Jain

**Affiliations:** ^1^ Department of Periodontology Dr. Harvansh Singh Judge Institute of Dental Sciences & Hospital, Panjab university Chandigarh Punjab India; ^2^ Faculty of Dentistry Meenakshi Academy of Higher Education and Research, Meenakshi Ammal Dental College and Hospital Chennai Tamil Nadu India; ^3^ Dr. D.Y. Patil Dental College & Hospital, Dr. D.Y. Patil Vidyapeeth Pune Maharashtra India

**Keywords:** gingivitis, mouthwash, octenidine, oral microbial load, plaque

## Abstract

**Objective:**

Octenidine dihydrochloride is an antimicrobial cationic surfactant compound. We conducted a systematic review to determine the efficacy of octenidine‐based mouthwash on plaque formation, gingivitis, and oral microbial growth in subjects with or without periodontal disease.

**Materials and Methods:**

PubMed/MEDLINE, ScienceDirect, Google Scholar, and Cochrane Library were searched for relevant studies. The review was conducted per PRISMA guidelines. Only randomized controlled trials and observational studies comparing octenidine with placebo or other mouthwashes in healthy subjects with or without periodontal disease, were considered for this review. The endpoints included percentage reduction in plaque index (PI), gingival index (GI), absolute reduction in the mean number of colony‐forming units (CFU/ml [log_10_]) and adverse effects (AEs; tooth staining/mucosal tolerance).

**Results:**

Ten randomized controlled and six observational studies fulfilled the selection criteria. Twice or thrice daily rinsing with 0.1% octenidine for 30–60 s produced significant reduction in plaque, gingivitis and oral microbial growth. Compared to control mouthwash or baseline, 0.1% octenidine inhibited plaque formation by ~38.7%–92.9%, which was either equal or greater than that of chlorhexidine gluconate. 0.1% octenidine reduced gingivitis by ~36.4%–68.37% versus control mouthwash or baseline and microbial growth by 0.37–5.3 colony‐forming units (vs. chlorhexidine: 0.4–4.23 colony‐forming units). Additional benefits of 0.1% octenidine were significant reduction in the number of bleeding sites, papilla bleeding index, sulcus bleeding index, and gingival fluid flow.

**Conclusion:**

Within the limitations of this study, there exists moderate evidence that 0.1% OCT was found to be an effective antiplaque agent. Octenidine inhibited plaque formation upto 93% and gingivitis upto 68% versus placebo and was either superior or comparable to chlorhexidine. Octenidine was well‐tolerated and safe and can be an effective alternative to CHX and other contemporary mouthwashes.

## 1 INTRODUCTION

Dental caries and periodontal disease are common oral conditions, though frequently neglected, affect ~3.58 billion individuals worldwide (Vos et al., 2017). Although mechanical methods of plaque control (tooth brushing or flossing) are reasonably effective in maintaining oral hygiene, these alone are insufficient for complete plaque removal, especially in inaccessible areas of the oral cavity (Arora et al., 2014). Therefore, a chemical means to achieve optimum oral hygiene such as daily use of an antimicrobial mouthwash is recommended, particularly in individuals who are at risk of developing periodontitis (Barnett, 2006). Unlike a toothpaste, mouthwash being liquid can significantly reduce total oral microbial load as it rinses the entire oral cavity including inaccessible areas and soft and hard oral surfaces (Ciancio, 2015). To maintain the oral hygiene antimicrobial mouthwash is useful in older age people, patients who are unable to brush their teeth. It is particularly useful in the maintenance of oral hygiene in patients unable to brush their teeth due to illness or surgery, or in the elderly and in patients with special needs (Prasad et al., 2016). Commercial mouthwashes have antimicrobial and breath‐freshening properties, and contain a combination of antiseptics, astringents, breath fresheners, essential oils (EOs), flavorings, and so on (Sykes et al., 2016).

Chlorhexidine gluconate (CHX) is a widely used, time‐tested agent in mouthwashes effective against plaque formation, gingivitis, and oral microbial growth (Van Strydonck et al., 2012). However, the associated side‐effects such as tooth discoloration, mucosal irritation, supragingival calculus formation, cytotoxicity, and taste alteration limit its benefits when it is required to be used for the long term. A novel antimicrobial cationic surfactant compound, octenidine dihydrochloride (OCT) was developed at the Sterling‐Winthrop Research Institute, Rensselaer, NY in the 1980s (Al‐Doori et al., 2007; Slee & O'Connor, 1983). OCT disrupts the cell membrane of fungi, bacteria, and yeast through strong adherence to lipid components and binding to negatively charge microbial surfaces (Brill et al., 2006; Kodedová & Sychrová, 2017). Data indicate that systemic absorption following cutaneous or oral administration of OCT is negligible. However, no data have been reported for secondary pharmacodynamics, drug interactions, metabolism and microbial resistance (Al‐Doori et al., 2007; EPAR, 2009). It is mainly eliminated in the feces, no accumulation in the body has been reported (EPAR, 2009). 0.1% OCT mouthwash with excipients, additives and flavorings is approved for maintaining oral hygiene in pre‐ and post‐periodontal or oral surgical interventions, gingivitis treatment, gingival bleeding upon probing and halitosis prevention (Sykes et al., 2016).

With a broad spectrum of activity, OCT is effective against several bacterial strains and fungi in vitro (Bailey et al., 1984). It acts at an extremely broad range of pH (1.6–12.2; Ellabib et al., 1990). While comparing the antimicrobial activity, OCT was found to be more effective than CHX, polyvinylpyrrolidone‐iodine (PVP‐I), polyhexamethylene biguanide, and triclosan in an in‐vitro study (Koburger et al., 2010). Additionally in a randomized cross‐over study, OCT was more efficacious than EOs, acriflavine hydrochloride, cetylpyridinium digluconate, and hydrogen peroxide in reducing the oral aerobic bacterial growth (Pitten & Kramer, 1999). Octenidine was superior to CHX and alexidine in inhibiting plaque‐forming enzymes in the oral cavity in an in vitro study (Bailey et al., 1984). Importantly, OCT was less cytotoxic than CHX, EOs or PVP‐I against gingival fibroblasts and epithelial cells in an in vitro study (Schmidt et al., 2016). In an in vitro study, octenidine effectively inhibits colony‐forming microbe co‐aggregation thereby preventing plaque formation (Smith et al., 1991).

Although several clinical studies have reported antibacterial and antiplaque efficacy of OCT against established antimicrobial mouthwashes, there are no systematic evaluations on the effectiveness of OCT‐based mouthwash. Therefore, this review systematically evaluated the evidence on the effectiveness of 0.1% OCT mouthwash, either as a monotherapy or as an adjunct, to regular oral hygiene against plaque formation, gingivitis and oral microbial load in subjects with or without periodontal disease.

## 2 METHODS

We did a systematic search based on the standard procedure adhering to PICO‐research question, where population considered is healthy subjects with or without periodontal diseases, intervention is octenidine and comparison is with placebo or other mouthwashes, outcomes are reduction in plaque (PI) and gingival index (GI), absolute reduction in the mean number of colony‐forming units (CFU/ml [log_10_]) and AEs (tooth staining/mucosal tolerance). Multiple biomedical literature databases were searched to identify relevant literature based on the Preferred Reporting Items for Systematic Reviews and Meta‐Analyses (PRISMA) guidelines (Moher et al., 2015).

### 2.1 Objectives

The primary objective was to determine the effectiveness of OCT mouthwash alone or as an adjunct to regular oral hygiene in controlling plaque, gingivitis, and oral microbial growth against a control mouthwash or only regular oral hygiene in subjects with/without periodontal disease. The secondary objective was to compare OCT versus CHX mouthwash in plaque, gingivitis and oral microbial growth reduction. Adverse effects (AEs) associated with OCT mouthwash use were also identified. The endpoints included percentage reduction in plaque and gingival index (GI), absolute reduction in the mean number of colony‐forming units (CFU/ml [log_10_]) for cariogenic bacteria and AEs (tooth staining/mucosal tolerance).

### 2.2 Inclusion and exclusion criteria

Only randomized controlled trials (RCTs) and observational studies that compared any concentration of OCT against a control mouthwash/mouthwashes containing CHX, EOs or PVP‐I in healthy subjects with or without periodontal disease, were included. Preclinical studies, case series and patents were excluded.

### 2.3 Search strategy

A comprehensive search using combinations of the terms “octenidine,” “plaque,” “gingivitis,” and “antimicrobial efficacy” was performed on PubMed/MEDLINE, ScienceDirect, Google Scholar, and the Cochrane Library to identify the literature published up to February 2019. Complete details of search strategies and all search results for a given combination of search terms, and their relevance to the study are provided as Appendix.

### 2.4 Screening and selection

The titles, abstracts and methods of all studies identified in the searches were inspected. At this stage, the selection was over‐inclusive to minimize the risk of missing relevant studies. The full text of all relevant studies was obtained if search keywords were found either in the title and abstract. Relevant data from the abstracts of studies for which full texts could not be obtained or the full text was not available in English, were also included. Snowball searches of the reference lists of all studies and reviews were also conducted to potentially identify relevant studies. Only those studies fulfilling all selection criteria were considered for data extraction.

### 2.5 Quality assessment

The quality of the methodologies of all included studies were assessed per modified Jadad scale criteria (Jadad et al., 1996), risk of bias assessment tool (Cochrane Collaboration for RCTs; Higgins et al., 2011), and the Newcastle‐Ottawa scale (observational studies; Deeks et al., 2003).

### 2.6 Data extraction

Two independent reviewers extracted the data including the study type, pharmacological agents used for intervention and their concentration, treatment duration, study population, sample size, number of dropouts, participants' age, gender, plaque and gingivitis scores and oral bacterial CFUs. The data of additional parameters, if reported, were also extracted: bleeding sites, papilla bleeding index (PBI), sulcus bleeding index (SBI) and pocket depth (PD) scores. Mean values, standard errors of the mean and standard deviations (SD) were extracted for all outcomes, including statistical significance levels. Oral microbial growth was expressed as CFU/ml (log_10_).

### 2.7 Ethics Statement

No ethical approval per se was needed for this work as data only from the previous published studies in which informed consent and/or ethical approval was duly obtained by primary investigators had been systematically reviewed and analyzed. No human subject participants are directly involved in the conduct of this work.

## 3 RESULTS

### 3.1 Search results

**FIGURE 1:**
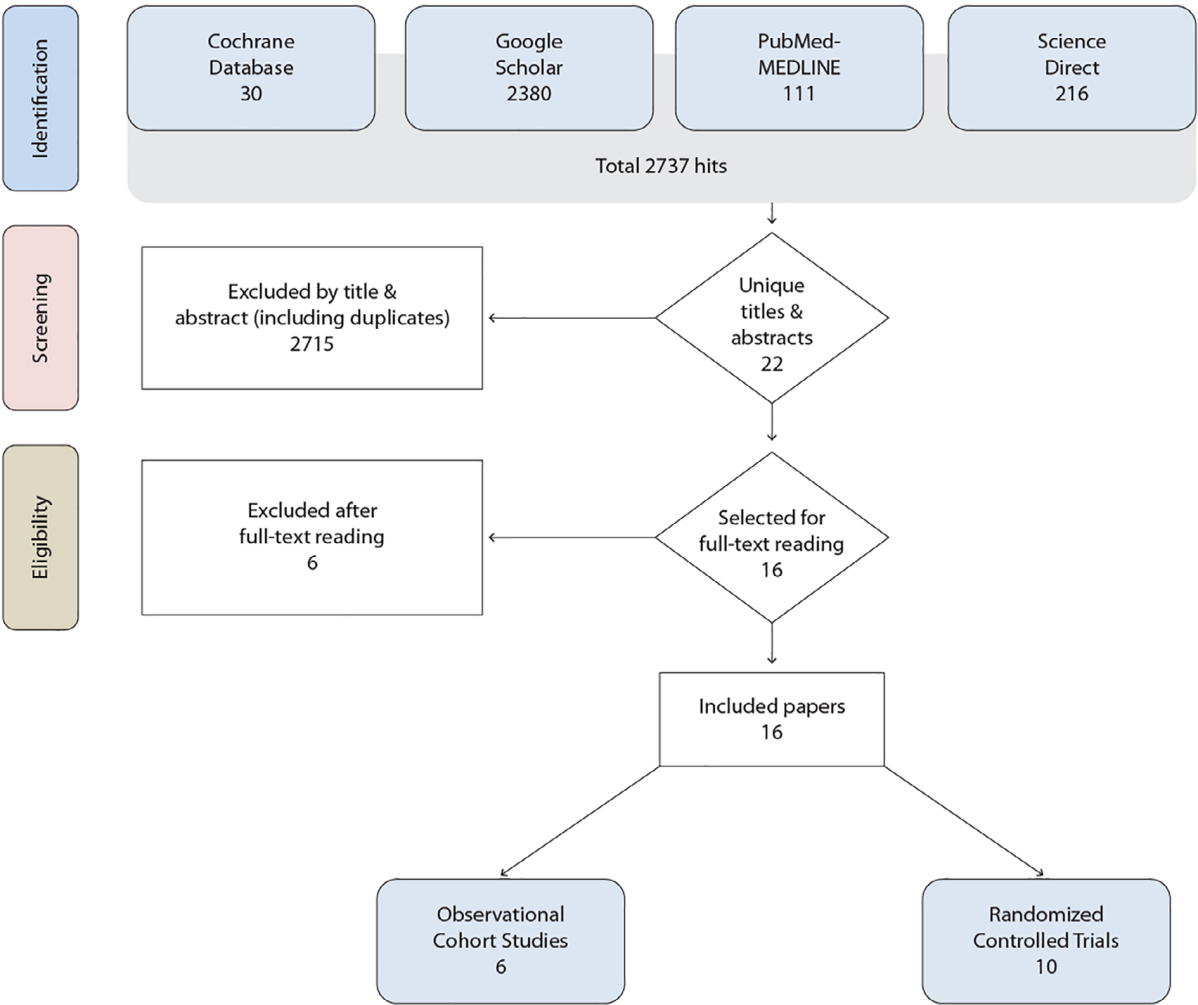
Flow chart of screening and selection of studies

Electronic searches yielded 2737 hits; 2721 studies were rejected (not meeting selection criteria; Figure 1). Inter‐observer agreement of a Cohen's kappa (*K*) of 0.77 was achieved for selection of studies. Overall, 16 relevant studies, 10 RCTs (Beiswanger et al., 1990; Hemanth et al., 2017; Jain et al., 2017; Koertge et al., 1986; Lobene et al., 1985; Lorenz et al., 2018; Mutters et al., 2015; Patters et al., 1983, 1986; Welk et al., 2016) and six observational studies (Dogan et al., 2008, 2009; Gušić et al., 2016; Kocak et al., 2009; Kramer et al., 1998; Pitten & Kramer, 1999) were processed for data extraction.

### 3.2 Study characteristics

Detailed study characteristics are presented in Table 1. There was a considerable heterogeneity in the design of the included studies. Overall, OCT was used twice or thrice daily with 30–60 s of rinsing. The number, gender, age and type of participants, and dentifrice use varied considerably among the studies. Ten studies enrolled healthy adults, two included children with orthodontic appliances; and four studies included children with poor oral hygiene, HIV patients with periodontal disease, adults with periodontal pockets, and adults with gingivitis, respectively. We classified the studies as those assessing the short‐term (immediately post‐rinsing up to 120 min, *n* = 4) and long‐term (2 days to 3 months, *n* = 12) effects of OCT on study outcomes.

**TABLE 1:** Clinical characteristics of studies included in the review

Study	Study type	Study population (*N*)	Sample size (*n*)	Study dropouts (*n*)	Treatment	Findings
Beiswanger et al. (1990)	Double‐blind randomized controlled trial	Adult volunteers (451)	0.1% OCT (226) Placebo (225)	0.1% OCT (90) Placebo (48)	30 s twice daily for 90 days	Compared to placebo, rinsing with OCT showed significantly less plaque (39%), gingivitis (50%), and bleeding sites (60%), but significantly higher stain formation and longer prophylaxis times for stain removal
Dogan et al. (2008)	Observational	Patients with fixed orthodontic appliances (18)	0.1% OCT 0.2% CHX 7.5% PVP‐I Physiologic Saline	–	Rinsing with one of the mouthwashes for 30 s	Compared with CHX and PVP‐I, OCT had significantly greater inhibitory effect on total and cariogenic bacteria (*S*. *mutans* and *L* species) from 15 to 120 min post‐application (*p* < 0.01)
Dogan et al. (2009)	Observational	Patients with fixed orthodontic appliances (18)	0.1% OCT 0.2% CHX 7.5% PVP‐I Physiologic Saline	–	15 ml of the mouthwash for 30 s once daily for 5 days	Compared with CHX and PVP‐I, OCT had significantly greater inhibitory effect on total and cariogenic bacteria (*S*. *mutans* and *Lactobacillus* species) from beginning of study until fifth day post‐bonding of orthodontic appliances (*p* < 0.1)
Gušić et al. (2016)	Randomized study	HIV +ve patients with periodontal disease (60)	0.1% OCT + Periodontal therapy (30) Periodontal therapy only (30)	–	15 ml twice daily for 7 days	Compared with periodontal therapy alone, OCT had a pronounced decrease in PBI and PD at 1‐month F/U and greater increase in PD at 3 months post treatment. OCT eliminated atypical microorganisms within 1 month post treatment
Hemanth et al. (2017)	Double‐blind randomized controlled trial	Adults with periodontal pockets (4–6 mm) (30)	0.1% OCT (15) 0.2% CHX (15)	–	21 days (irrigation procedure once daily at night for 14 days and maintained upto 21 days without irrigation)	Compared with CHX, OCT showed highly significant reduction of mean SBI until day 21, and significant improvement in PI by day 7 (*p* < 0.001). Both groups showed significant PD reduction and CAL gain from day 0 to day 21
Jain et al. (2017)	Double‐blind randomized controlled trial	Child patients (6–12 years) (84)	Pts. with poor oral hygiene (Control): 0.1% OCT (21) 0.2% CHX (21) Pts. with ALL + chemotherapy (Test): 0.1% OCT (21) 0.2% CHX (21)	Control: 0.2% CHX (1) Test: 0.2% CHX (1)	10 ml of the mouthwash for 1 min twice a day for 5 days	OCT showed statistically significant (*p* < 0.001) decrease in bacterial colony count, and was more effective than CHX in reducing salivary microbial load
Kocak et al. (2009)	Observational	Healthy adult volunteers (27)	0.1% OCT (9) 0.12% CHX (9) Antimicrobial enzymatic rinse (9)	–	Rinsing with mouthwash for 2 min	OCT had a significant effect on *S*. *mutans* and preserved antimicrobial efficiency even after 60 min. OCT was found to be more effective than other mouth rinses over *S*. *mutans* (*p* < 0.05)
Koertge et al. (1986)	Randomized controlled trial	Subjects with excellent oral hygiene (88)	0.1% OCT (22) 0.1% OCT in aq. Soln. (22) Mouthwash VEH (22) 0.1% OCT + VEH (22)	22	Thrice daily for 21 days	OCT inhibited formation of supra‐gingival plaque. Compared to VEH, GI was significantly lower in all OCT groups (*p* < 0.05) at 2 and 3 weeks of rinsing. OCT was effective in prevention of dental plaque formation and gingivitis over a 3‐week period in the absence of other means of oral hygiene
Kramer et al. (1998)	Observational	Healthy volunteers	0.1% OCT 0.2% CHX 0.025% Cetylpyridinium chloride 0.2%Acriflavine‐soln.	–	Not clear from abstract	OCT had significantly stronger impact on microbial burden of oral cavity than CHX immediately and 10 min post‐application
Lobene et al. (1985)	Double‐blind randomized controlled trial	Adult male subjects (61)	0.1% OCT in VEH 0.1% OCT in water 0.05% OCT in VEH Control VEH	–	One minute twice daily rinsing for 7 days	Compared to control VEH, all OCT formulations significantly reduced plaque accumulation (*p* = 0.0001), and crevicular fluid flow (*p* = 0.04) in the absence of other means of oral hygiene. In VEH group, Gingival inflammation increased more compared to other groups. Group. Tooth staining was observed in all groups, but was non‐significant
Lorenz et al. (2018)	Randomized, evaluator blinded, Incomplete, crossover trial	Adult patients with gingivitis (90)	0.1% OCT (29) 0.15% OCT (30) 0.2% OCT (31)	–	10 ml, 30 s twice daily in the morning and in the evening for 4 days	Significant differences in SBC reduction and mean GI/PIs between all OCT concentrations and placebo observed (*p* < 0.001). Adverse effects increased with increasing OCT concentrations
Mutters et al. (2015)	Double‐blind randomized controlled trial	Adult patients (139) is ventilated cardiothoracic surgical patients and patients with hemato‐oncological malignancies requiring stem cell transplantation	Ventilated cardiothoracic surgical pts. (Stratum i Surgical): 0.1% OCT (41) 0.2% CHX (50) Medical Pts. with hemato‐oncological malignancies (Stratum ii): 0.1% OCT (25) 0.2% CHX (23)	30	Four times daily for 7 days	Compared with CHX and Macrogol, OCT showed highly significant reduction of oropharyngeal flora in both study groups
Patters et al. (1983)	Double‐blind randomized controlled trial	Healthy adult males (60)	0.1% OCT in VEH 0.1% OCT in aq. solution 0.05% OCT in VEH VEH only	4	15 ml of mouthwash for 1 min twice daily for 7 days	Compared with VEH, OCT almost completely inhibited plaque formation over 7 days in the absence of other oral hygiene measures. Compared to control group, gingival fluid flow was significantly lower in all OCT groups. Tooth stain increased in all groups using OCT, however the stain was easily removed by polishing with a rubber cup and pumice
Patters et al. (1986)	Double‐blind randomized controlled trial	Healthy adult volunteers (88)	0.1% OCT in VEH (TID) (22) 0.1% OCT in VEH (BID) (22) 0.1% OCT in water (TID) (22) VEH only (TID) (22)	24	15 ml for 60 s, twice (OCT in VEH) or three times daily for 21 days	Compared with VEH, BID and TID 0.1% OCT groups showed significantly less plaque accumulation and gingivitis at days 7, 14, and 21
Pitten and Kramer (1999)	Cross over study	Healthy adult volunteers (10)	0.1% OCT 0.2% CHX Essential oil, and so on	–	20 ml mouthwash for 30 s	0.1% OCT exerted sustained antimicrobial efficacy of >1.0 log colony forming units 1 h post‐application
Welk et al. (2016)	Double‐blind randomized controlled trial	Healthy adult volunteers (16)	0.1% OCT 0.12% CHX Essential Oils Placebo	–	20 ml mouthwash for 1 min twice daily for 4 days	OCT and CHX showed significantly lower bacterial counts on the tooth surface and oral mucosa compared to placebo

Abbreviations: ALL, acute lymphoblastic leukemia; Aq., aqueous; BID, twice daily; CAL, clinical attachment level; CHX, chlorhexidine; F/U, follow‐up; GI, gingival index; L, Lactobacillus; OCT, octenidine; PBI, papilla bleeding index; PD, probing pocket depth; PI, plaque index; PVP‐I, polyvinylpyrrolidone‐iodine complex; *S*. *mutans*, *Streptococcus mutans*; SBI, sulcus bleeding index; TID, thrice daily; VEH, vehicle; Pt, patient.

### 3.3 Quality assessment

Table 2 represents the estimated risk of bias of the included RCTs. In five RCTs (Beiswanger et al., 1990; Lorenz et al., 2018; Patters et al., 1983, 1986; Welk et al., 2016), the potential risk of bias was low. The risk was moderate for three RCTs (Hemanth et al., 2017; Jain et al., 2017; Mutters et al., 2015); and it was unclear for two studies that were available only as abstracts (Koertge et al., 1986; Lobene et al., 1985). Only five RCTs clearly the described adequate random sequence generation and allocation concealment, while seven reported adequate blinding of study participants. Seven RCTs adequately reported withdrawal rates by number and reason per arm (Table 3). Table 4 presents the quality assessment of the six observational studies. The overall quality of two studies was good (Gušić et al., 2016; Pitten & Kramer, 1999); and that of three, was fair (Dogan et al., 2008, 2009; Kocak et al., 2009). One study could not be appraised as the full text was unavailable in English (Kramer et al., 1998).

**TABLE 2:** Risk of bias assessment of trials of octenidine as a mouthwash

Study	Random sequence generation	Allocation concealment	Selective reporting	Other sources of bias	Adequate blinding of personnel and participants	Blinding (outcome assessment)	Incomplete outcome data
Beiswanger et al. (1990)	Low	Low	Low	Low	Low	Low	Low
Hemanth et al. (2017)	Unclear	Low	Low	Unclear	Low	Low	Unclear
Jain et al. (2017)	Low	Low	Low	Unclear	Low	Low	Unclear
Koertge et al. (1986)	Unclear	Unclear	Unclear	Unclear	Unclear	Unclear	Low
Lobene et al. (1985)	Unclear	Unclear	Unclear	Unclear	Unclear	Unclear	Unclear
Lorenz et al. (2018)	Low	Low	Low	Low	Low	Low	Low
Mutters et al. (2015)	Low	Unclear	Low	Unclear	Unclear	Unclear	Low
Patters et al. (1983)	Unclear	Low	Low	Low	Low	Low	Low
Patters et al. (1986)	Low	Low	Low	Low	Low	Low	Low
Welk et al. (2016)	Low	Low	Low	Low	Low	Low	Low

**TABLE 3:** Quality assessment of trials of octenidine as a mouthwash based on the modified Jadad scale

No.	Question	Beiswanger et al. (1990)	Hemanth et al. (2017)	Jain et al. (2017)	Koertge et al. (1986)	Lobene et al. (1985)	Lorenz et al. (2018)	Mutters et al. (2015)	Patters et al. (1983)	Patters et al. (1986)	Welk et al. (2016)
1	Is this a RCT study?	Yes	Yes	Yes	Yes	Yes	Yes	Yes	Yes	Yes	Yes
2	Reported as randomized	Yes	Yes	Yes	Yes	Yes	Yes	Yes	Yes	Yes	Yes
3	Randomization is appropriate	Yes	Yes	Yes	ND	ND	Yes	Yes	ND	Yes	Yes
4	Double blinding is reported	Yes	Yes	Yes	ND	Yes	Yes	Yes	Yes	Yes	Yes
5	Double blinding is appropriate	Yes	Yes	Yes	ND	ND	Yes	ND	Yes	Yes	Yes
6	Withdrawals are reported by number and reason per arm	Yes	No	Yes	Yes	No	Yes	Yes	Yes	Yes	No
7	Jadad Score (/5)	5	4	5	3	2	5	4	4	5	4
8	Method(s) used to assess adverse events is described	Yes	No	Yes	Yes	Yes	Yes	No	Yes	Yes	No
9	Method(s) of statistical analysis is described	Yes	Yes	Yes	Yes	No	Yes	Yes	Yes	Yes	Yes
10	Inclusion and/or exclusion of the requirements is reported	Yes	Yes	Yes	No	No	Yes	Yes	Yes	Yes	Yes
11	Modified Jadad score (/8)	8	6	8	5	3	8	6	7	8	6
12	Was the allocation adequately concealed?	Yes	Yes	Yes	Unclear	Unclear	Yes	Unclear	Yes	Yes	Yes
13	Was the analysis based on intention to treat principle?	Unclear	Unclear	No	Unclear	Unclear	Yes	Unclear	Unclear	Unclear	Unclear
14	Was the sample size justified?	Unclear	Unclear	Unclear	Unclear	Unclear	Yes	Yes	Unclear	Unclear	Unclear

Abbreviations: ND, not described; RCT, randomized controlled trial.

**TABLE 4:** Quality assessment of cohort studies of octenidine as a mouthwash based on the Newcastle–Ottawa scale

Items on the checklist	Dogan et al. (2008)	Dogan et al. (2009)	Gušić et al. (2016)	Kocak et al. (2009)	Pitten and Kramer (1999)
Representativeness of the exposed cohort	Selected group	Selected group	Truly representative	Truly representative	Truly representative
Selection of the non‐exposed cohort	ND	ND	Drawn from the same community as the exposed cohort	Drawn from the same community as the exposed cohort	Drawn from the same community as the exposed cohort
Ascertainment of exposure	Secure record	Secure record	Secure record	Secure record	Secure record
Demonstration that outcome of interest was not present at start of study	Yes	Yes	Yes	Yes	Yes
The study controls for age, sex and marital status	No	No	No	Yes	No
Study controls for other factors	Yes	Yes	Yes	Yes	Yes
Assessment of outcome	Self‐report	Self‐report	Independent blind assessment	Self‐report	Self‐report
Was follow‐up long enough for outcomes to occur	Yes	Yes	Yes	No	Yes
Adequacy of follow‐up of cohorts	Complete follow up‐ all subjects accounted for	Complete follow up‐ all subjects accounted for	Complete follow up‐ all subjects accounted for	Complete follow up‐ all subjects accounted for	Complete follow up‐ all subjects accounted for
Overall quality of the study	Fair quality	Fair quality	Good quality	Fair quality	Good quality

Abbreviation: ND, not described.

### 3.4 Plaque formation

Data regarding the effect of OCT on plaque formation were available for nine studies (Table 5; Beiswanger et al., 1990; Hemanth et al., 2017; Koertge et al., 1986; Lobene et al., 1985; Lorenz et al., 2018; Patters et al., 1983, 1986; Welk et al., 2016; Gušić et al., 2016). These OCT mouthwashes were of different concentrations (0.1%, 0.15%, 0.2%); and all significantly reduced plaque formation.

**TABLE 5:** Effect on plaque index (PI)

Study	Reduction in PI
Beiswanger et al. (1990)	After 3 months of treatment with 0.1% OCT, there was a 38.7% reduction in the PI compared to placebo mouth rinse, and difference between means was significant at *α* = 0.05
Gušić et al. (2016)	0.1% OCT (periodontal therapy + OCT mouthwash for 7 days) showed 48.61% and 47.22% reduction in the PI at 1 and 3 months, respectively, compared to baseline (*p* < 0.01)
Hemanth et al. (2017)	Compared to baseline, mouth rinsing with 0.1% OCT reduced PI by 51.08%, 56.71% and 56.71% at days 7, 14, and 21, respectively. Plaque inhibition was greater with 0.1% OCT than 0.2% CHX on day 7 (51.08% vs. 33.46%, *p* = 0.001), although both were equally effective on days 14 (56.71% vs 53.85%, *p* = 0.064) and 21 (56.71% vs. 61.54%, *p* = 1.0)
Lorenz et al. (2018)	Compared to placebo (0.9% saline solution), reduction in PI by 67.09%, 72.78%, and 73.42% was seen with 0.1%, 0.15% and 0.2% OCT mouthwashes, respectively. Comparisons were statistically significant (*p* < 0.001) compared to all OCT concentrations (ANOVA)
Patters et al. (1983)	Seven days of rinsing with 0.1% OCT mouthwash reduced PI by 70.29% compared to placebo mouthwash (vehicle without OCT; *p* < 0.01). The 0.05% OCT mouthwash was also reduced PI but the reduction was lower than 0.1% OCT
Patters et al. (1986)	Compared to placebo mouthwash (vehicle without OCT), 0.1% OCT mouthwash (twice a day) reduced PI by 79.63%, 88.49%, and 90.53% at days 7, 14, and 21 (*p* < 0.000001). When used thrice daily, they reduced PI by 83.33%, 89.21%, and 92.9% (*p* < 0.000001)
Welk et al. (2016)	Compared to placebo (contained 0.5% Tween® 20, 0.05% [v/v] peppermint oil [Minthea peperita, Lavita] and 0.005% food coloring solution [green]), 0.1% OCT mouth rinse reduced PI by 47.66% (*p* < 0.0001), and 0.12% CHX mouthwash reduced PI by 57.87% (*p* < 0.0001)^.^. Difference between reductions by OCT and CHX were not statistically significant (*p* = 0.682)
Lobene et al. (1985)	Only the abstract were available and required quantitative details were not available from the abstracts
Koertge et al. (1986)	

Abbreviations: CHX, chlorhexidine; OCT, octenidine; PI, plaque index.

Five studies compared the effects of OCT against a control mouthwash; three enrolled healthy volunteers (Beiswanger et al., 1990; Patters et al., 1983, 1986); one, adults with gingivitis (Lorenz et al., 2018); and one, HIV patients with periodontal disease (Gušić et al., 2016). Compared to control mouthwash or baseline, OCT inhibited plaque formation by ~38.7%–92.9% within 4 days to 3 months of use (Beiswanger et al., 1990; Lorenz et al., 2018; Patters et al., 1983, 1986).

Two studies compared the effectiveness of OCT and CHX on plaque growth (Hemanth et al., 2017; Welk et al., 2016). In one study, rinsing twice daily, after breakfast/evening for 4 days produced similar plaque growth inhibition with 0.1% OCT versus 0.12% CHX (47.66% vs. 57.87%, *p* = 0.682; Welk et al., 2016). In another study, 1 ml of 0.1% OCT/0.2% CHX was injected into the periodontal pocket of the affected tooth to treat localized periodontitis (unlike other studies requiring rinsing with mouthwash). Plaque inhibition was greater with 0.1% OCT than 0.2% CHX on day 7 (51.08% vs. 33.46%, *p* = 0.001), although both were equally effective on days 14 and 21 (Hemanth et al., 2017).

### 3.5 Gingivitis

The effect of OCT on GI was evaluated in six studies (Table 6; Beiswanger et al., 1990; Koertge et al., 1986; Lobene et al., 1985; Lorenz et al., 2018; Patters et al., 1986; Gušić et al., 2016). All studies reported a significant reduction in GI with OCT versus control mouthwash (Table 6). When compared to control mouthwash or baseline, the percentages of GI reduction ranged from ~36.4% to 68.37% from day 4 to 3 months of use. None of the eligible studies included in review reported the comparative efficacy of OCT versus CHX on GI.

**TABLE 6:** Effect on gingival index (GI)

Study	Reduction in GI
Beiswanger et al. (1990)	After 3 months of treatment 0.1% OCT reduced GI by 50% compared to placebo mouth rinse, difference between means was significant (*α* = 0.05)
Gušić et al. (2016)	0.1% OCT (periodontal therapy + OCT mouthwash for 7 days) showed 65.27% and 67.07% reduction in GI at 1 and 3 months, respectively compared to the baseline (*p* < 0.01)
Lorenz et al. (2018)	Compared to placebo (0.9% saline solution), GI was reduced by 41.07%, 64.4%, 59.25% with 0.1%, 0.15% and 0.2% OCT mouthwashes. Comparisons were statistically significant (*p* < 0.001) compared to all OCT concentrations (ANOVA)
Patters et al. (1986)	Compared to placebo mouthwash (vehicle without OCT), 0.1% OCT mouthwash twice a day could reduce GI by 58.63%, 67.86%, 68.37% at days 7, 14, and 21, respectively. When used thrice daily, it could reduce GI by 63.79%, 65.48% and 67.35% (*p* < 0.000001)
Lobene et al. (1985)	As only the abstracts were available, the required quantitative details could not be stated here
Koertge et al. (1986)	

Abbreviations: GI, gingival index; OCT, octenidine.

Twice or thrice daily rinsing with 0.1% OCT significantly inhibited gingivitis versus control mouthwash in the absence of oral hygiene measures (brushing; Patters et al., 1986). A significant decrease in GI by 65.27% at 1 month and 67.07% at 3 months from baseline was observed in a study by Gušić et al. (2016). Octenidine also demonstrated a favorable effect on GI in studies by Lobene et al. (1985) and Koertge et al. (1986).

### 3.6 Oral microbial growth

The effect of OCT on oral microbial growth was assessed in 10 studies (Table 7; Pitten & Kramer, 1999; Jain et al., 2017; Lorenz et al., 2018; Mutters et al., 2015; Welk et al., 2016; Dogan et al., 2008, 2009; Gušić et al., 2016; Kocak et al., 2009; Kramer et al., 1998), and all reported significant reductions in the total oral microbial growth (*p* < 0.05 to *p* < 0.001).

**TABLE 7:** Effect of 0.1% Octenidine on oral microbial growth

Study	Reduction in total bacterial count
Dogan et al. (2008)	Rinsing with 0.1% OCT and 0.2% CHX mouthwashes, respectively, reduced the total bacterial count (log_10_ CFU/ml) by 4.4 and 1 at 15 min; 3.9 and 1.6 at 30 min; 3.7 and 1.3 at 60 min; and 3.1 and 1.9 at 120 min (all, *p* < 0.001). Control mouthwash (physiological saline) did not reduce the total bacterial count at any time points
Dogan et al. (2009)	Rinsing with 0.1% OCT, 0.2% CHX and control (physiological saline) mouthwashes, respectively, reduced the total bacterial count (log_10_ CFU/ml) by 4.43, 0.96 and 0.049 at 15 min; 3.23 and 2.04 at day 2; 4.13, 2.14, and −0.04 at day 3; 3.73, 1.9 and −0.05 at day 5. These reductions were statistically significant for OCT and CHX (*p* < 0.001) and for control the *p* was 0.041
Gušić et al. (2016)	Reduction in total bacterial count (log_10_ CFU/ml) from baseline was 5.29 and 5.3 for 0.1% OCT (periodontal therapy + OCT mouthwash), and 5.35 and 5.44 for control (periodontal therapy only) at 1 and 3 months, respectively (*p* < 0.01)
Jain et al. (2017)	Reduction in *S*. *mutant* count (log_10_ CFU/ml) from baseline was 3.95 and 4.11 for 0.2% CHX and 0.1% OCT groups (*p* = 0.430), respectively at day 1. Corresponding values at day 3 was 4.18 and 4.32 (*p* = 0.916); and at day 5 were 4.23 and 4.36 (*p* = 0.121). Reduction in *Lactobacillus* count (log_10_ CFU/ml) from baseline was 3.30, 3.36 for 0.2% CHX and 0.1% OCT groups (*p* = 1.000), respectively at day 1. Corresponding values at day 3 were 3.41 and 3.46 (*p* = 0.743); and at day 5 were 3.48 and 3.51 (0.725)
Lorenz et al. (2018)	At day 4, reduction in total bacterial count (log_10_ CFU/ml) from baseline was 0.37, 0.86, and 1.13 with 0.1%, 0.15% and 0.2% OCT mouthwashes, respectively. The corresponding value for placebo (0.9% saline solution) was −0.75. Difference from placebo was statistically significant for all OCT concentrations
Mutters, Neubert, Nieth, & Mutters et al. (2015)	Rinsing with 0.1% OCT and 0.2% CHX mouthwashes, respectively, reduced the total bacterial count (log_10_ CFU/ml) by 1.3 and 4.1 (*p* = 0.04) at day 3; 1 and 5 (*p* = 0.003) at day 7
Pitten and Kramer (1999)^$^	The 0.1% OCT, 0.2% CHX and control (distilled sterile water) mouthwashes, respectively, reduced the total bacterial count (log_10_ CFU/ml) by 2.15, 1.41 and 0.36, 10 min after rinsing; and 1.96, 1.52 and 0.09, 30 min after rinsing; and −0.06, 1.73,1.38 and −0.06 60 min after rising
Welk et al. (2016)	On the tooth surface, reduction in total bacterial count (log_10_ CFU/ml) from baseline on day 1 was 0.7 with both 0.1% OCT and 0.12% CHX, and it was 0.2 with placebo (0.5% Tween® 20, 0.05% [v/v] peppermint oil [Minthea peperita, Lavita] and 0.005% food coloring solution [green]). The corresponding values at day 5 were 0.4 and −2.9. Reduction in total mucosal bacterial count from baseline (log_10_ CFU/ml) was 1.6, 1.9, 0.3 with 0.1% OCT, 0.12% CHX and placebo, respectively, on day 1. Corresponding values on day 5 were 0.6, 0.6 and −2.8. Difference between placebo and OCT (*p* = 0.003 for tooth surface and *p* < 0.0001 for mucosal surface) and/or CHX (*p* < 0.0001 for tooth surface and *p* = 0.001 for mucosal surface) were statistically significant but the difference between OCT and CHX was not significant (*p* = 0.781 for tooth surface and *p* = 1 for mucosal surface)
Kramer et al. (1998)	The required quantitative details were not available from the published articles by Kocak et al., and the abstract by Kramer et al.
Kocak et al. (2009)	

Abbreviations: CFU, colony forming unit; CHX, chlorhexidine; OCT, octenidine.

Two studies assessed the short‐term effects (≤120 min) of OCT mouthwash on microbial growth versus placebo (Dogan et al., 2008; Pitten & Kramer, 1999). The reduction from baseline in the microbial growth was 1.73–4.4 CFU/ml (log_10_) versus no change to −0.06 CFU/ml (log_10_) with placebo (Dogan et al., 2008; Pitten & Kramer, 1999). Additionally, growth of cariogenic bacteria *S*. *mutans* (0.1% OCT vs. placebo: 0 min, 4.6 vs. 4.6; 15 min, 0 vs. 4.6; 120 min, 2.9 vs. 4.66; *p* < 0.001) and *Lactobacillus* species (0.1% OCT vs. placebo: 0 min, 4.17 vs. 4.07; 15 min, 0 vs. 4.3; 120 min, 1 vs. 4.43; *p* < 0.001) was significantly inhibited (Dogan et al., 2008). Four studies assessed the long‐term effects (2 days to 3 months) of OCT‐based versus control mouthwash on microbial growth (Dogan et al., 2009; Gušić et al., 2016; Lorenz et al., 2018; Welk et al., 2016). OCT reduced the microbial growth from baseline by 0.37–5.3 CFU/ml (log_10_) from day 1 to 3 months of rinsing. In HIV‐positive patients with periodontal disease, the bacterial count in the subgingival plaque samples was not significantly different between 0.1% OCT and periodontal therapy alone (5.3 vs. 5.44 CFU/ml [log_10_], *p* > 0.05) at 3 months (Gušić et al., 2016). Interestingly, no atypical microorganisms were observed 1 month post‐treatment in HIV‐positive patients receiving 0.1% OCT (*p* < 0.05); whereas these were detected in 34.5% of patients in the control group. At 3 months, *Prevotella intermedia* was not found in any patient rinsing with 0.1% OCT but was isolated from 4.8% of controls (Gušić et al., 2016).

Eight studies compared the efficacies of OCT and CHX on oral microbial growth (Dogan et al., 2008, 2009; Jain et al., 2017; Kocak et al., 2009; Kramer et al., 1998; Mutters et al., 2015; Pitten & Kramer, 1999; Welk et al., 2016); seven studies reported OCT having superior efficacy over CHX, while one (Welk et al., 2016) reported comparable efficacy. Two studies assessed the short‐term effects (≤120 min) of OCT on microbial growth versus CHX (Dogan et al., 2008; Kramer et al., 1998). The reduction from baseline in microbial growth was 1.73–4.4 CFU/ml (log_10_) with OCT versus 1.0–1.9 CFU/ml (log_10_) with CHX in short‐term. The long‐term reduction in the CFU/ml (log_10_) ranged from 0.4 to 5.0 with OCT versus 0.4–4.23 with CHX up to 3 months of use (Dogan et al., 2009; Mutters et al., 2015; Welk et al., 2016).

The total bacterial count (3.73 vs. 1.9 CFU/ml [log_10_]) significantly reduced for 0.1% OCT given in vivo to patients with fixed orthodontic appliances versus 0.2% CHX, from the baseline until the fifth day post‐bonding of the appliance. A similar pattern of inhibition in the growth of *Lactobacillus* species and *S*. *mutans* was observed (Dogan et al., 2009). A significantly stronger impact of OCT in reducing oral microbial load than CHX, immediately and 10 min after application was seen in a study by Kramer et al., 1998 (Kramer et al., 1998). It was observed that 0.1% OCT was more effective than 0.12% CHX in reducing *S*. *mutans* growth at 1, 10, and 60 min after rinsing (Kocak et al., 2009).

The antimicrobial efficacy of 0.1% OCT versus 0.2% CHX was determined in pediatric patients with poor oral hygiene (control, *n* = 42), and with acute lymphocytic leukemia (test, *n* = 42). The test and control groups were divided into two subgroups, 0.1% OCT and 0.2% CHX. A significantly greater decrease in the mean CFU of oral bacteria, including *S*. *mutans* (0.1% OCT vs. 0.2% CHX: 4.36 vs. 4.23 CFU/ml; *p* < 0.001) and *Lactobacillus* species (0.1% OCT vs. 0.2% CHX: 3.51 vs. 3.48 CFU/ml; *p* < 0.001) was observed with 0.1% OCT than 0.2% CHX (Jain et al., 2017). A significant reduction in mean CFUs from baseline after 7 days of rinsing for 30 s four times daily with 0.1% OCT versus 0.1% CHX (5.0 vs. 1.0; *p* = 0.003), was observed in ventilated cardiothoracic surgical patients and in patients with hemato‐oncologic malignancies (Mutters et al., 2015).

### 3.7 Additional benefits of OCT‐based mouthwashes

The number of bleeding sites significantly reduced (60%) after 3 months of 0.1% OCT use versus control mouthwash with routine toothbrushing with a dentifrice (Beiswanger et al., 1990). In HIV‐positive patients with periodontal disease, a significant decrease in mean PBI (1.22 ± 0.85 vs. 0.22 ± 0.7; *p* < 0.01) and PD scores (0.27 ± 0.40 vs. 0.30 ± 0.30) was seen with OCT versus placebo at 3 month follow‐up (Gušić et al., 2016). Both OCT and CHX significantly reduced PD scores and clinical attachment levels at follow‐up, OCT significantly reduced the mean SBI (0.13 ± 0.34 vs. 0.73 ± 0.59, mean difference: 0.60 ± 0.25; *p* = 0.002) at 21 days (Hemanth et al., 2017). It was observed that 0.1% OCT in vehicle (day 0 vs. day 7: 25 vs. 21; *p* < 0.05) and 0.1% OCT in aqueous solution (day 0 vs. day 7: 31 vs. 21; *p* < 0.05), significantly reduced gingival fluid flow (periotron units) versus vehicle alone (day 0 vs. day 7: 26 vs. 38) after 7 days following twice‐daily rinsing (Patters et al., 1983). All formulations containing different concentrations of OCT significantly reduced crevicular fluid flow after 7 days of twice‐daily rinsing (Lobene et al., 1985). Thus, 0.1% OCT effectively prevented mucositis in susceptible patients (Mutters et al., 2015; Table 1).

### 3.8 Adverse effects of OCT‐based mouthwashes

Tooth stain was a common non‐serious AE associated with OCT use, reported in six studies (Table 8; Beiswanger et al., 1990; Koertge et al., 1986; Lobene et al., 1985; Lorenz et al., 2018; Patters et al., 1983, 1986). In five studies, subjects refrained from oral hygiene measures, including tooth brushing during the test period. Tooth staining was reversible following single tooth brushing with a dentifrice or polishing with a rubber cup or pumice (Beiswanger et al., 1990; Koertge et al., 1986; Lorenz et al., 2018; Patters et al., 1983, 1986). AEs increased with increasing OCT concentrations (Lorenz et al., 2018).

**TABLE 8:** Adverse effects of 0.1% octenidine

Adverse effect with 0.1% OCT	Beiswanger et al. (1990)	Kramer et al. (1998)	Koertge et al. (1986)	Lobene et al. (1985)	Lorenz et al. (2018)	Patters et al. (1983)	Patters et al. (1986)	Number of studies reporting AE
Dental stain	+	−	+	+	+	+	+	6
Diminution of taste	−	−	−	−	−	−	+	1
Burning Sensation	+	−	+	−	−	−	−	2
Nausea	+	−	−	−	−	−	−	1
Erythema/Irritation	+	−	+	−	−	+	+	4
Blisters/Ulcerations	+	−	+	−	−	−	+	3
Swelling	+	−	+	−	−	−	−	2
Hives	+	−	+	−	−	−	−	2
Tongue discoloration	−	−	−	−	+	−	+	2
Inflammation	−	−	−	−	−	+	+	2
Tingling of tongue	−	−	−	−	−	+	+	2

Abbreviations: +, present; −, absent; AE, adverse events; OCT, octenidine.

The other reported AEs were decreased taste, tongue dorsum discoloration, bitter aftertaste, mild tingling of the tongue, and poor mucosal tolerance with varied incidence across the included studies (Table 8). A higher proportion of subjects using OCT in aqueous solution experienced mucosal intolerance in the studies by Koertge et al., 1986; Patters et al., 1983; Patters et al., 1986; however, the OCT formulation in vehicle was well tolerated by the oral mucosa and no significant AEs were observed.

## 4 DISCUSSION

Oral health is an integral part in general health. Thus, to enjoy optimum quality of life it is essential to maintain a better oral health status without gingivitis, periodontitis, tooth decay and tooth loss. The fundamental objective of periodontal therapy is subgingival plaque control. Since the mechanical removal of subgingival plaque is time‐consuming and technically demanding, use of adjunctive antimicrobial agents such as mouthwash is a simple and effective option for optimum oral hygiene (Tartaglia et al., 2017). OCT, being positively charged, exerts its bactericidal action by binding to the negatively charged bacterial cell membranes and to soft and hard oral surfaces. It disrupts the phospholipid bilayer and attacks the enzyme systems causing the cell wall to lose integrity and leak its cytoplasmic contents. OCT exhibits high affinity for cardiolipin, a lipid exclusively present in the bacterial cell membranes and therefore damages bacterial cells leaving the epithelium unaffected (EPAR, 2009). Efficacy of OCT depends upon its concentration, bacterial load, and duration of contact with the bacteria.

This is a first‐of‐its‐kind systematic review evaluating evidence on the effectiveness of OCT‐based mouthwashes. Although the included studies have investigated the effects of a 0.1% OCT‐based mouthwash in subjects with or without periodontal diseases, most studies were small when considered independently. Collectively, the efficacy of a 0.1% OCT‐based mouthwash against plaque, gingivitis, and oral microbial growth irrespective of treatment duration and mechanical oral hygiene, has been demonstrated (Table 1).

All studies assessing effects of a 0.1% OCT mouthwash reported significant decrease in plaque formation versus control mouthwash. Moreover, the effect of twice‐daily rinsing was observed even after short‐term use for 4 days in some studies, and a long‐term use for up to 3 months in others. A significant reduction in GI following the use of 0.1% OCT‐based mouthwash versus control mouthwash was reported in all studies, except one; and 10 studies reported a significant reduction in the total oral microbial growth. The effectiveness of 0.1% OCT‐based mouthwashes in HIV‐positive patients suggests that this formulation can be favorably used in patients with comorbid diseases in addition to chronic periodontitis.

In the presence of toothbrushing, but without toothpaste, 0.1% OCT significantly reduced cariogenic bacterial growth including *S*. *mutans* and *Lactobacillus* species versus control mouthwash (Jain et al., 2017). Moreover, 0.1% OCT prevented oral microbial growth for 12–16 h after the last rinsing (Welk et al., 2016). Complete elimination of *P*. *intermedia*, a periodontal pathogen, was reported following a twice‐daily week‐long rinsing with 0.1% OCT after periodontal therapy (mechanical debridement) and toothbrushing (Gušić et al., 2016). Additionally, antibacterial effect of 0.1% OCT in the saliva was observed even during the suspension of toothbrushing. Since the placement of orthodontic appliances, especially brackets and wires, obstructs maintenance of effective oral hygiene by mechanical means, rinsing with a 0.1% OCT mouthwash can maintain adequate hygiene in plaque‐infected sites, especially around the bracket bases that protects tooth enamel integrity, prevents white spot lesion, and periodontal damage (Dogan et al., 2009). Additional benefits of 0.1% OCT use included a significant reduction in the number of bleeding sites (Beiswanger et al., 1990). Further, significant reduction in PBI with 0.1% adjunct OCT versus periodontal therapy alone suggests a pronounced reduction in inflammation with the former (Gušić et al., 2016).

Data comparing the efficacies of 0.1% OCT and 0.2% CHX mouthwashes, were obtained from eight studies (Dogan et al., 2008, 2009; Jain et al., 2017; Kocak et al., 2009; Kramer et al., 1998; Mutters et al., 2015; Pitten & Kramer, 1999; Welk et al., 2016). Rinsing with 0.1% OCT versus 0.2% CHX had a pronounced plaque‐reducing effect (Welk et al., 2016). No study compared the effects of 0.1% OCT‐ versus 0.2% CHX‐based mouthwashes on GI. In all studies comparing the efficacy of 0.1% OCT with 0.2% CHX mouthwashes, the former was more effective than the latter in its antibacterial effect. Compared to most mouthwashes with effects lasting 15–30 min post‐rinsing, the 0.1% OCT‐based mouthwash exerted its effects even after 120 min (Dogan et al., 2008). A significant reduction in oropharyngeal flora with 0.1% OCT than 0.2% CHX was seen in ventilated cardiothoracic surgical patients and in patients with hemato‐oncological malignancies (Mutters et al., 2015). Also, 0.1% OCT was more effective than 0.2% CHX on the fifth day of use even in the absence of toothbrushing or brushing without toothpaste (*p* < 0.001; Jain et al., 2017; Dogan et al., 2009). Hence, a 0.1% OCT‐based mouthwash is an effective alternative to CHX and other contemporary mouthwashes in maintaining optimal oral health.

Tooth staining was a commonly reported AE following use of 0.1% OCT, as most studies refrained subjects from oral hygiene measures during the study; however, stain removal with single toothbrushing was reported. A majority of subjects claimed to continue use of the 0.1% OCT‐based formulation (Lorenz et al., 2018). Despite the fact that bitter taste and mucosal irritation caused by OCT in aqueous solutions was a significant concern in earlier studies, OCT formulations in a mouthwash vehicle (at all concentrations) were well‐tolerated by the oral mucosa and no significant AEs were observed. Overall, OCT is a chemically stable formulation with a low toxicity profile, is easy and safe to handle, nonflammable, and well‐tolerated in clinical use (Assadian, 2016).

### 4.1 Limitations

Although the 0.1% OCT‐based mouthwash was efficacious in maintaining optimal oral hygiene for a short duration in all studies, evidence supporting long‐term use is yet to be established. The sample size of all included studies was small and a study by Kramer et al. reporting on the microbial effects could not be accessed as a full text manuscript, thus supposes a careful interpretation and extrapolation to a larger population.

## 5 CONCLUSIONS

Within the limitation of this systematic review, there is moderate quality of evidence that 0.1% OCT was found to be an effective antiplaque agent, as weighed on evidence based Grade recommendations (Guyatt et al., 2008). OCT was efficacious, and substantially reduced plaque formation, gingivitis and oral microbial growth. It was more effective than placebo and other common chemical agents used for plaque control. OCT was either superior or comparable to CHX‐based mouthwashes in controlling dental plaque. Furthermore, it was also found to be effective in controlling gingivitis in patients with fixed orthodontic appliances. The use of 0.1% OCT mouthwash resulted in complete elimination of atypical oral microbe species, even at 1 month after therapy. Additional benefits included prevention of white spot lesions. OCT was well‐perceived, tolerable, safe, and an effective alternative to CHX and other contemporary antibacterial mouthwashes. However, further studies assessing the long‐term effects of a 0.1% OCT‐based mouthwash, involving larger sample size, are required to confirm the results.

## CONFLICT OF INTEREST

Authors state no conflict of interest.

## AUTHOR CONTRIBUTIONS

Vishakha Grover, Jaideep Mahendra, Dharmrajan Gopalakrishnan, Ashish Jain: conceptualization and methodology; Vishakha Grover, Jaideep Mahendra, Dharmrajan Gopalakrishnan, Ashish Jain: data acquisition and interpretation; Vishakha Grover: supervision or oversighting research activity plan and execution; Vishakha Grover, Jaideep Mahendra, Dharmrajan Gopalakrishnan, Ashish Jain: reviewing, editing and finalization of manuscript. All authors meet the International Committee of Medical Journal Editors (ICMJE) criteria for authorship of this manuscript; take the complete responsibility for accuracy of the content presented in manuscript.

## Supporting information

**Appendix** S1: Supporting Information

## Data Availability

The data that supports the findings of this study are available in the supplementary material of this article.
