# Supplementary material for: Effect of octenidine mouthwash on plaque, gingivitis, and oral microbial growth: A systematic review
Source: Clin Exp Dent Res. 2021 Jul 6;7(4):450–64. doi: 10.1002/cre2.386 (PMC8404485; doi:10.1002/cre2.386)
Supplement: Supplementary file 1 — Appendix S1: Supporting Information [file CRE2-7-450-s001.docx]

**Octenidine Dihydrochloride as a Mouthwash – Systematic Literature Review**

**Search Terms:**

1. Octenidine and Plaque
2. Octenidine and Gingivitis
3. Octenidine and Antimicrobial Efficacy

**Electronic Databases:**

1. Cochrane Database of Systematic Reviews (CDSR)
2. Google Scholar
3. PubMed/MEDLINE
4. ScienceDirect

**Databases Searched, Search Terms, Corresponding Number of Search Hits, Relevant & Irrelevant References**

| **Database** | **Search Terms** | **Total No. of Hits** | **Relevant References (No.)** | **Irrelevant References (No.)** |
| --- | --- | --- | --- | --- |
| CDSR | Octenidine and Plaque | 15 |  |  |
|  | Octenidine and Gingivitis | 12 |  |  |
|  | Octenidine and Antimicrobial Efficacy | 3 | 0 | 3 |
| Google Scholar | Octenidine and Plaque | 1000 | 14 | *In vitro* studies=125  *Ex vivo* studies =7  On skin=19  In primates=7  In rodents=5  In pigs=1  In dogs=1  Reviews=106  Non-RCT=92  Non-Octenidine=99  Patents=516  Duplicates=8 |
|  | Octenidine and Gingivitis | 380 | 12 | *In vitro* studies=95  *Ex vivo* studies=2  In primates=4  In rodents=3  In dogs=2  On skin=14  Reviews=70  Non-RCT=85  Non-Octenidine=93 |
|  | Octenidine and Antimicrobial Efficacy | 1000 | 11 | *In vitro* studies=311  *Ex vivo* studies=18  In primates=3  In pigs=1  In dogs=2  In rodents=5  Reviews=176  Non-RCT=246  Non-Octenidine=103  Not as mouthwash=124 |
| PubMed / MEDLINE | Octenidine and Plaque | 19 | 7 | *In vitro* studies=4 In primates=3 In rodents=1 Reviews=2 Non-RCT=1 |
|  | Octenidine and Gingivitis | 9 | 4 | Not as mouthwash=1 In primates=2 Reviews=1 Non-RCT=1 |
|  | Octenidine and Antimicrobial Efficacy | 83 | 3 | *In vitro* studies=43  *Ex vivo* studies=1  In rodents=2  Reviews=3  Non-RCT=3 Not as mouthwash=28 |
| ScienceDirect | Octenidine and Plaque | 47 | 2 | *In vitro* studies=9  In hamsters=1  Reviews=5  Non-RCT=28 Not as mouthwash=2 |
|  | Octenidine and Gingivitis | 23 | 2 | *In vitro* studies=6  Reviews=2  Non-RCT=12 Not as mouthwash=1 |
|  | Octenidine and Antimicrobial Efficacy | 146 | 1 | *In vitro* studies=24  In rodents=1  Reviews=40  Non-RCT=52 Not as mouthwash=24  Non-Octenidine=4 |

**Biomedical Literature Databases Search Results**

**I] Cochrane Database of Systematic Reviews (CDSR)** **Search Results**

i. **Octenidine and Plaque** (Search conducted at 6:00 AM EST, 21/02/2019)

1. Study to Assess the Inhibition of Plaque Formation in Subjects with a Gingival Index ≤1.5. NCT03378401. Https://clinicaltrials.gov/show/nct03378401, **2017** | added to CENTRAL: 31 May 2018 | 2018 Issue 5. **Relevance:** YES
2. Study to Assess the Inhibition of Plaque Formation in Subjects With a Gingival Index ≤1.5. NCT03322124. Https://clinicaltrials.gov/show/nct03322124, **2017** | added to CENTRAL: 31 May 2018 | 2018 Issue 5. **Relevance:** YES
3. The clinical effects of a mouthrinse containing 0.1% octenidine. BB Beiswanger, ME Mallatt, MS Mau, RD Jackson, DK Hennon. Journal of dental research, 1990, 69(2), 454‐457 | added to CENTRAL: 31 January 1998 | 1998 Issue 1. **Relevance:** YES
4. Antibacterial and antiplaque efficacy of a commercially available octenidine-containing mouthrinse. A Welk, M Zahedani, C Beyer, A Kramer, G Müller. Clinical oral investigations, 2016, 20(7), 1469‐1476 | added to CENTRAL: 31 October 2017 | 2017 Issue 10. **Relevance:** YES
5. Impact of different concentrations of an octenidine dihydrochloride mouthwash on salivary bacterial counts: a randomized, placebo-controlled cross-over trial. K Lorenz, Y Jockel-Schneider, N Petersen, P Stolzel, M Petzold, U Vogel, T Hoffmann, U Schlagenhauf, B Noack. Clinical oral investigations, 2018, 22(8), 2917‐2925 | added to CENTRAL: 30 November 2018 | 2018 Issue 11. **Relevance:** YES
6. Effects of octenidine mouthrinse on plaque formation and gingivitis in humans. MR Patters, J Nalbandian, FC Nichols, CE Niekrash, JE Kennedy, RA Kiel. J-periodontal-res, 1986, 21(2) | added to CENTRAL: 31 October 1998 | 1998 Issue 4. **Relevance:** YES
7. The effect of octenidine on plaque, gingivitis and crevicular fluid. RR Lobene, PM Soparkar, M Tavares, MB Newman. Journal of dental research, 1985, 64(Spec), 235, Abstract no: 542 | added to CENTRAL: 31 October 2011 | 2011 Issue 4. **Relevance:**
8. Inhibition of plaque formation in humans by octenidine mouthrinse. MR Patters, K Ånerud, CL Trummel, KS Kornman, J Nalbandian, PB Robertson. Journal of periodontal research, 1983, 18(2), 212‐219 | added to CENTRAL: 30 June 2013 | 2013 Issue 6. **Relevance:** YES
9. Prevention of plaque and gingivitis formation by octenidine hydrochloride. TE Koertge, KG Palcanis, HA Schenkein, CJ Cavallaro. Journal of dental research, 1986, 65(Spec [AADR Abstracts]), 246, Abstract no: 691 | added to CENTRAL: 31 October 2011 | 2011 Issue 4. **Relevance: Yes**
10. Inhibition of plaque formation in humans by octenidine mouthrinse. MR Patters, K Ånerud, CL Trummel, KS Kornman, J Nalbandian, PB Robertson. Journal of periodontal research, 1983, 18(2), 212‐219 | added to CENTRAL: 30 June 2013 | 2013 Issue 6. **Relevance:** NO (duplicate)
11. Measurements of chlorhexidine, p-chloroaniline, and p-chloronitrobenzene in saliva after mouth wash before and after operation with 0.2% chlorhexidine digluconate in maxillofacial surgery: a randomised controlled trial. H Below, O Assadian, R Baguhl, U Hildebrandt, B Jager, K Meissner, DJ Leaper, A Kramer. British journal of oral & maxillofacial surgery, 2016, (no pagination) | added to CENTRAL: 28 February 2017 | 2017 Issue 2. **Relevance:** YES
12. Measurements of chlorhexidine, p-chloroaniline, and p-chloronitrobenzene in saliva after mouth wash before and after operation with 0.2% chlorhexidine digluconate in maxillofacial surgery: a randomised controlled trial. H Below, O Assadian, R Baguhl, U Hildebrandt, B Jager, K Meissner, DJ Leaper, A Kramer. British journal of oral & maxillofacial surgery, 2017, (no pagination) | added to CENTRAL: 30 June 2017 | 2017 Issue 6. **Relevance:** NO (duplicate)
13. Treatment of Periodontal Disease with an Octenidine-based Antiseptic in HIV-positive Patients. I Gusic, D Medic, M Radovanovic Kanjuh, M Duric, S Brkic, V Turkulov, T Predin, J Mirnic. International journal of dental hygiene, 2016, 14(2), 108‐116 | added to CENTRAL: 30 November 2017 | 2017 Issue 11. **Relevance:**? (Study involves HIV patients)
14. Measurements of chlorhexidine, p-chloroaniline, and p-chloronitrobenzene in saliva after mouth wash before and after operation with 0.2% chlorhexidine digluconate in maxillofacial surgery: a randomised controlled trial. H Below, O Assadian, R Baguhl, U Hildebrandt, B Jager, K Meissner, DJ Leaper, A Kramer. British journal of oral & maxillofacial surgery, 2017, (no pagination) | added to CENTRAL: 28 February 2017 | 2017 Issue 2. **Relevance:** NO (duplicate)
15. Treatment of Periodontal Disease with an Octenidine-based Antiseptic in HIV-positive Patients. I Gusic, D Medic, M Radovanovic Kanjuh, M Duric, S Brkic, V Turkulov, T Predin, J Mirnic. International journal of dental hygiene, 2016, 14(2), 108‐116 | added to CENTRAL: 30 November 2017 | 2017 Issue 11. **Relevance:** NO (duplicate)

**ii. Octenidine and Gingivitis** (Search conducted at 6:50 AM EST, 21/02/2019)

1. Dose Finding Study to Assess Octenidine Mouthwash Concentrations in Comparison to Placebo. NCT02138552. Https://clinicaltrials.gov/show/nct02138552, 2013 | added to CENTRAL: 31 May 2018 | 2018 Issue 5. **Relevance:** YES
2. The clinical effects of a mouthrinse containing 0.1% octenidine. BB Beiswanger, ME Mallatt, MS Mau, RD Jackson, DK Hennon. Journal of dental research, 1990, 69(2), 454‐457 | added to CENTRAL: 31 January 1998 | 1998 Issue 1. **Relevance:** YES
3. Study to Assess the Inhibition of Plaque Formation in Subjects with a Gingival Index ≤1.5. NCT03378401. Https://clinicaltrials.gov/show/nct03378401, **2017** | added to CENTRAL: 31 May 2018 | 2018 Issue 5. **Relevance:** YES
4. Study to Assess the Inhibition of Plaque Formation in Subjects With a Gingival Index ≤1.5. NCT03322124. Https://clinicaltrials.gov/show/nct03322124, **2017** | added to CENTRAL: 31 May 2018 | 2018 Issue 5. **Relevance:** YES
5. Impact of different concentrations of an octenidine dihydrochloride mouthwash on salivary bacterial counts: a randomized, placebo-controlled cross-over trial. K Lorenz, Y Jockel-Schneider, N Petersen, P Stolzel, M Petzold, U Vogel, T Hoffmann, U Schlagenhauf, B Noack. Clinical oral investigations, 2018, 22(8), 2917‐2925 | added to CENTRAL: 30 November 2018 | 2018 Issue 11. **Relevance:** YES
6. Effects of octenidine mouthrinse on plaque formation and gingivitis in humans. MR Patters, J Nalbandian, FC Nichols, CE Niekrash, JE Kennedy, RA Kiel. J-periodontal-res, 1986, 21(2) | added to CENTRAL: 31 October 1998 | 1998 Issue 4. **Relevance:** YES
7. The effect of octenidine on plaque, gingivitis and crevicular fluid. RR Lobene, PM Soparkar, M Tavares, MB Newman. Journal of dental research, 1985, 64(Spec), 235, Abstract no: 542 | added to CENTRAL: 31 October 2011 | 2011 Issue 4. **Relevance:**
8. Prevention of plaque and gingivitis formation by octenidine hydrochloride. TE Koertge, KG Palcanis, HA Schenkein, CJ Cavallaro. Journal of dental research, 1986, 65(Spec [AADR Abstracts]), 246, Abstract no: 691 | added to CENTRAL: 31 October 2011 | 2011 Issue 4. **Relevance:**
9. Measurements of chlorhexidine, p-chloroaniline, and p-chloronitrobenzene in saliva after mouth wash before and after operation with 0.2% chlorhexidine digluconate in maxillofacial surgery: a randomised controlled trial. H Below, O Assadian, R Baguhl, U Hildebrandt, B Jager, K Meissner, DJ Leaper, A Kramer. British journal of oral & maxillofacial surgery, 2016, (no pagination) | added to CENTRAL: 28 February 2017 | 2017 Issue 2. **Relevance:** YES
10. Measurements of chlorhexidine, p-chloroaniline, and p-chloronitrobenzene in saliva after mouth wash before and after operation with 0.2% chlorhexidine digluconate in maxillofacial surgery: a randomised controlled trial. H Below, O Assadian, R Baguhl, U Hildebrandt, B Jager, K Meissner, DJ Leaper, A Kramer. British journal of oral & maxillofacial surgery, 2017, (no pagination) | added to CENTRAL: 30 June 2017 | 2017 Issue 6. **Relevance:** YES
11. Inhibition of plaque formation in humans by octenidine mouthrinse. MR Patters, K Ånerud, CL Trummel, KS Kornman, J Nalbandian, PB Robertson. Journal of periodontal research, 1983, 18(2), 212‐219 | added to CENTRAL: 30 June 2013 | 2013 Issue 6. **Relevance:** YES
12. Measurements of chlorhexidine, p-chloroaniline, and p-chloronitrobenzene in saliva after mouth wash before and after operation with 0.2% chlorhexidine digluconate in maxillofacial surgery: a randomised controlled trial. H Below, O Assadian, R Baguhl, U Hildebrandt, B Jager, K Meissner, DJ Leaper, A Kramer. British journal of oral & maxillofacial surgery, 2017, (no pagination) | added to CENTRAL: 28 February 2017 | 2017 Issue 2. **Relevance:** NO (duplicate)

**iii.Octenidine and Antimicrobial Efficacy** (Search conducted at 7:20 AM EST, 21/02/2019)

1. Topical antimicrobial agents for treating foot ulcers in people with diabetes. Jo C Dumville, Benjamin A Lipsky, Christopher Hoey, Mario Cruciani, Marta Fiscon, Jun Xia. Cochrane Systematic Review - Intervention Version published: 14 June 2017. **Relevance:** NO (Octenidine is not used as a mouthwash)
2. Dressings and topical agents for treating venous leg ulcers. Gill Norman, Maggie J Westby, Amber D Rithalia, Nikki Stubbs, Marta O Soares, Jo C Dumville. Cochrane Systematic Review - Intervention Version published: 15 June 2018. **Relevance:** NO (Octenidine is not used as a mouthwash)
3. Antiseptics for burns. Gill Norman, Janice Christie, Zhenmi Liu, Maggie J Westby, Jayne M Jefferies, Thomas Hudson, Jacky Edwards, Devi Prasad Mohapatra, Ibrahim A Hassan, Jo C Dumville. Cochrane Systematic Review - Intervention Version published: 12 July 2017. **Relevance:** NO (Octenidine is not used as a mouthwash)

**II] Google Scholar Search Results** (Search conducted at 7:40 AM EST, 21/02/2019)

(Only unique relevant studies not found in other databases are included)

1. Short-term relative antibacterial effect of octenidine dihydrochloride on the oral microflora in orthodontically treated patients. AA Dogan, AK Adiloglu, S Onal, ES Cetin, E Polat, E Uskun, F Koksal. International Journal of Infectious Diseases. Volume 12, Issue 6, November 2008, Pages e19-e25
2. Antibacterial and antiplaque efficacy of a commercially available octenidine-containing mouthrinse. A Welk, M Zahedani, C Beyer, A Kramer, G Muller. Clinical Oral Investigations. September 2016, Volume 20, Issue 7, pp 1469–1476
3. Microbiological evaluation of octenidine dihydrochloride mouth rinse after 5 days' use in orthodontic patients. AA Dogan, ES Cetin, E Hüssein, AK Adiloglu - The Angle Orthodontist, 2009
4. Comparison of the efficacy of three different mouthrinse solutions in decreasing the level of streptococcus mutans in saliva. MM Kocak, S Ozcan, S Kocak, O Topuz, H Erten. Eur J Dent. 2009 Jan; 3(1): 57–61
5. Microbiological evaluation of 0.2% chlorhexidine gluconate mouth rinse in orthodontic patients. E Sari, I Birinci - The Angle Orthodontist, 2007. Vol. 77, No. 5, pp. 881-884.
6. The role of Octenidol®, Glandomed® and chlorhexidine mouthwash in the prevention of mucositis and in the reduction of the oropharyngeal flora: a double-blind randomized controlled trial. Mutters NT, Neubert TR, Nieth R, Mutters R. GMS Hyg Infect Control. 2015;10:Doc05. Published 2015 Feb 13. doi:10.3205/dgkh000248
7. Antimicrobial efficacy of antiseptic mouthrinse solutions. Pitten FA, Kramer A. Eur J Clin Pharmacol. 1999 Apr;55(2):95-100.
8. Anti-bacterial efficacy of octenidine as a mouth wash. Malhotra A, Bali A , Bareja R. International Journal Of Pharmaceutical Sciences And Research. 2016; Vol. 7(1): 340-344.
9. To evaluate the efficacy of sub gingival irrigation with octenidol and chlorhexidine on periodontal inflammation. Hemanth RK, Krishna V, Gundala R, Anusha G, Nishanthoury S, Rao MM. International Journal Of Pharmaceutical Sciences And Research, 2017, 5(3), 1920-1926.
10. The antimicrobial efficacy of 0.1 % Octenidine dihydrochloride and 0.2% Chlorhexidine gluconate mouthwash in children undergoing chemotherapy for Acute Lymphoblastic Leukemia. Jain A, Pandey RK, Mishra A. Rama Univ J Dent Sci 2017 June;4(2):1-6.

**III] PubMed/MEDLINE Search Results**

**Octenidine and Antimicrobial Efficacy** (Search conducted at 7:50 AM EST, 21/02/2019)

(Only unique relevant studies not found in other databases are included)

1. [Antiseptic efficacy and acceptance of Octenisept computed with common antiseptic mouthwashes]. Kramer A, Höppe H, Krull B, Pitten FA, Rosenau S. Zentralbl Hyg Umweltmed. 1998 Feb;200(5-6):443-56. German.

**Studies included in systematic review**

1. The clinical effects of a mouthrinse containing 0.1% octenidine. BB Beiswanger, ME Mallatt, MS Mau, RD Jackson, DK Hennon. Journal of dental research, 1990, 69(2), 454‐457. **Status:** Completed, full-text available
2. Antibacterial and antiplaque efficacy of a commercially available octenidine-containing mouthrinse. A Welk, M Zahedani, C Beyer, A Kramer, G Müller. Clinical oral investigations, 2016, 20(7), 1469‐1476. **Status:** Completed, full-text available
3. Impact of different concentrations of an octenidine dihydrochloride mouthwash on salivary bacterial counts: a randomized, placebo-controlled cross-over trial. K Lorenz, Y Jockel-Schneider, N Petersen, P Stolzel, M Petzold, U Vogel, T Hoffmann, U Schlagenhauf, B Noack. Clinical oral investigations, 2018, 22(8), 2917‐2925. **Status:** Completed, full-text available
4. Effects of octenidine mouthrinse on plaque formation and gingivitis in humans. MR Patters, J Nalbandian, FC Nichols, CE Niekrash, JE Kennedy, RA Kiel. J-periodontal-res, 1986, 21(2),154-162. **Status:** Completed, full-text available
5. The effect of octenidine on plaque, gingivitis and crevicular fluid. RR Lobene, PM Soparkar, M Tavares, MB Newman. Journal of dental research, 1985, 64(Spec), 235, Abstract no: 542. **Status:** *Need full-text*
6. Inhibition of plaque formation in humans by octenidine mouthrinse. MR Patters, K Ånerud, CL Trummel, KS Kornman, J Nalbandian, PB Robertson. Journal of periodontal research, 1983, 18(2), 212‐219. **Status:** Completed, full-text available
7. Prevention of plaque and gingivitis formation by octenidine hydrochloride. TE Koertge, KG Palcanis, HA Schenkein, CJ Cavallaro. Journal of dental research, 1986, 65(Spec [AADR Abstracts]), 246, Abstract no: 691. **Status:** *Need full-text*
8. Treatment of Periodontal Disease with an Octenidine-based Antiseptic in HIV-positive Patients. I Gusic, D Medic, M Radovanovic Kanjuh, M Duric, S Brkic, V Turkulov, T Predin, J Mirnic. International journal of dental hygiene, 2016, 14(2), 108‐116. **Status:** Completed, full-text available
9. Short-term relative antibacterial effect of octenidine dihydrochloride on the oral microflora in orthodontically treated patients. AA Dogan, AK Adiloglu, S Onal, ES Cetin, E Polat, E Uskun, F Koksal. International Journal of Infectious Diseases. Volume 12, Issue 6, November 2008, Pages e19-e25. **Status:** Completed, full-text available
10. Microbiological evaluation of octenidine dihydrochloride mouth rinse after 5 days' use in orthodontic patients. AA Dogan, ES Cetin, E Hüssein, AK Adiloglu - The Angle Orthodontist, 2009, 79:766–772. **Status:** Completed, full-text available
11. Comparison of the efficacy of three different mouthrinse solutions in decreasing the level of streptococcus mutans in saliva. MM Kocak, S Ozcan, S Kocak, O Topuz, H Erten. Eur J Dent. 2009 Jan; 3(1): 57–61. **Status:** Completed, full-text available
12. The role of Octenidol®, Glandomed® and chlorhexidine mouthwash in the prevention of mucositis and in the reduction of the oropharyngeal flora: a double-blind randomized controlled trial. Mutters NT, Neubert TR, Nieth R, Mutters R. GMS Hyg Infect Control. 2015;10: 2196-5226. **Status:** Completed, full-text available
13. Antimicrobial efficacy of antiseptic mouthrinse solutions. Pitten FA, Kramer A. Eur J Clin Pharmacol. 1999 Apr;55(2):95-100. **Status:** *Need full-text*
14. [Antiseptic efficacy and acceptance of Octenisept computed with common antiseptic mouthwashes]. Kramer A, Höppe H, Krull B, Pitten FA, Rosenau S. Zentralbl Hyg Umweltmed. 1998 Feb;200(5-6):443-56. German. **Status:** *Need full-text*
15. To evaluate the efficacy of sub gingival irrigation with octenidol and chlorhexidine on periodontal inflammation. Hemanth RK, Krishna V, Gundala R, Anusha G, Nishanthoury S, Rao MM. International Journal Of Pharmaceutical Sciences And Research, 2017, 5(3), 1920-1926. **Status:** Completed, full-text available
16. The antimicrobial efficacy of 0.1 % Octenidine dihydrochloride and 0.2% Chlorhexidine gluconate mouthwash in children undergoing chemotherapy for Acute Lymphoblastic Leukemia. Jain A, Pandey RK, Mishra A. Rama Univ J Dent Sci 2017 June;4(2):1-6. **Status:** Completed, full-text available
